# Integrated evaluation of clinical, pathological and radiological prognostic factors in squamous cell carcinoma of the lung

**DOI:** 10.1371/journal.pone.0223298

**Published:** 2019-10-04

**Authors:** Kyowon Gu, Ho Yun Lee, Kyungjong Lee, Joon Young Choi, Sook Young Woo, Insuk Sohn, Hong Kwan Kim, Yong Soo Choi, Jhingook Kim, Jae Ill Zo, Young Mog Shim

**Affiliations:** 1 Department of Radiology and Center for Imaging Science, Samsung Medical Center, Sungkyunkwan University School of Medicine, Seoul, Korea; 2 Division of Pulmonary and Critical Care Medicine, Department of Medicine, Samsung Medical Center, Sungkyunkwan University School of Medicine, Seoul, Korea; 3 Department of Nuclear Medicine, Samsung Medical Center, Sungkyunkwan University School of Medicine, Seoul, Korea; 4 Biostatistics and Clinical Epidemiology Center, Samsung Medical Center, Seoul, Korea; 5 Department of Thoracic and Cardiovascular Surgery, Samsung Medical Center, Sungkyunkwan University School of Medicine, Seoul, Korea; Weill Cornell Medical College in Qatar, QATAR

## Abstract

**Objective:**

Little is known about prognostic factors for lung squamous cell carcinoma (SCC). We aimed to explore radiologic and clinical factors affecting prognosis and to compare the prognosis of both central and peripheral lung SCCs.

**Materials and methods:**

Radiologic, clinical, and pathologic profiles of surgically confirmed SCCs from 382 patients were retrospectively reviewed. Tumor location, enhancement, necrosis, the presence of obstructive pneumonitis/atelectasis and underlying lung disease were evaluated on chest CT examination. Age, pulmonary function, tumor marker, and cancer stage were also assessed. Univariate and multivariate Cox regression analyses were performed to identify any correlation to overall survival (OS) and disease-free survival (DFS). Hazard rate estimation and competing risk analysis were done to evaluate recurrence pattern.

**Results:**

The median follow-up period was 56.2 months. Tumors were located centrally in 230 patients (60.2%) and peripherally in 152 patients (39.8%). Age (*p* = 0.002, hazard ratio [HR] 1.03, 95% confidence interval [CI] = [1.01, 1.06]) and interstitial lung abnormalities (ILAs) (*p*<0.001, HR 5.41, 95% CI = [3.08, 9.52]) were associated with poor OS on multivariate analysis. ILAs also had a strong association to DFS (*p*<0.001, HR 4.25, 95% CI = [3.08, 9.52]). Central cancers had two peaks of local recurrence development at 15 and 60 months after surgery, and peripheral tumors showed rising curves for metastasis development at 60 months.

**Conclusions:**

CT-determined ILAs are a strong biomarker predicting poor outcome. Prognosis may not vary according to tumor location, but the two groups exhibited different recurrence patterns.

## Introduction

While considerable progress has been made on classification and prognostication for lung adenocarcinomas (ADCs) by the International Association for the Study of Lung Cancer, American Thoracic Society, and European Respiratory Society, comparatively little has been made with regard to lung squamous cell carcinomas (SCCs), which account for 25–30% of all lung cancers [1–4]. Recent advances in understanding molecular aberrations through comprehensive genotyping have led to the development of targeted agents for lung SCC [5,6]. Targeted agents such as fibroblast growth factor receptor (*FGFR*) inhibitors, phosphatidylinositol 3-kinase (*PIK3K*) inhibitors, insulin-like growth factor receptor 1 (*IGF1R*) monoclonal antibodies, and *SOX2* have been investigated in clinical trials based on molecular genotyping. However, most agents have failed due either to toxicity or lack of efficacy. Therefore, identification of prognosticators and development of effective therapy for SCC is needed [7].

SCCs of the lung are categorized as central and peripheral cancers according to their location, and they manifest different clinicopathologic features. Peripheral SCCs are generally detected at a lower stage with less lymph node metastasis [8]. Central SCCs follow the dysplasia-carcinoma sequence, but the pathogenesis of peripheral cancers remains unclear [8,9].

Several biomarkers have been suggested as histologic or genomic prognosticators for resected SCCs, but few have been investigated in the era of radiology [7]. Imaging phenotypes potentially contain more comprehensive information than genomics data. Thus, we aimed to find radiologic variables influencing prognosis and compared peripheral and central SCCs, focusing on the patterns and dynamics of recurrence based on tumor location.

## Materials and methods

### Study population

**Fig 1:**
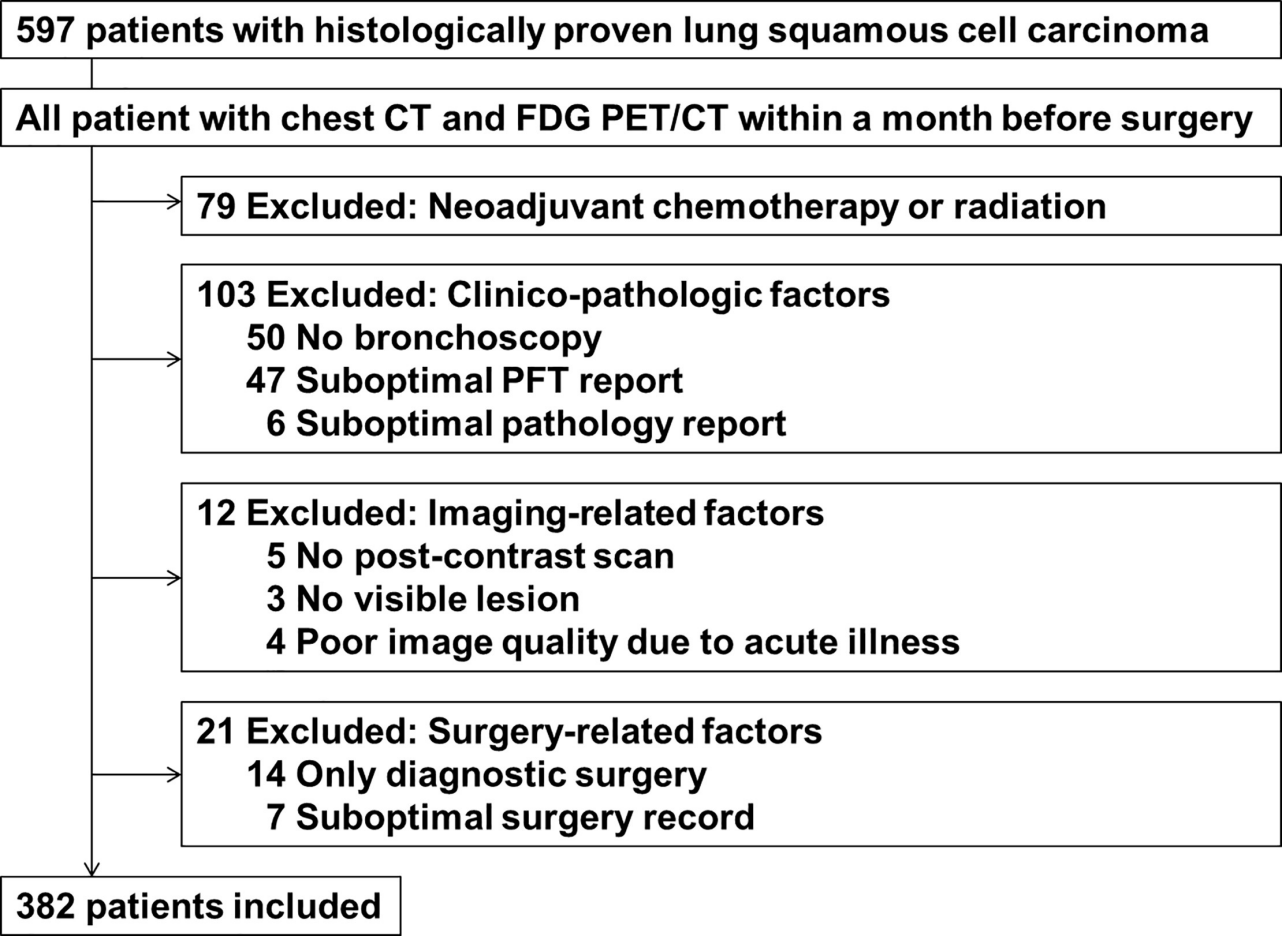
Flowchart of the enrollment process.

The Institutional Review Board of Samsung Medical Center approved this retrospective study (approval #2018-01-009-001), and the requirement for informed consent was waived. In total, 597 patients who had curative surgery for lung SCC at our institution between January 2003 and December 2012 were identified who also did not have metastasis on brain magnetic resonance imaging and whole body positron emission tomography/computed tomography (PET/CT) scans. We excluded 215 patients by the criteria presented in Fig 1. Ultimately, 382 patients (370 males, 12 females) were included. Adjuvant chemotherapy or radiation was initiated between one to two months after surgery. Clinical follow-up was done with routine chest CT scan once or twice a year per protocol.

### Image acquisition and analysis

We performed chest CT and 18F- Fluorodeoxyglucose (FDG) PET/CT scans within a month before surgery. CT scans were obtained using an 8- or 16-detector row CT scanner (LightSpeed16, GE Healthcare, Waukesha, WI) and the following parameters: detector collimation, 0.625 mm; field of view, 34.5 cm; beam pitch, 1.35 or 1.375; gantry speed, 0.6 seconds per rotation; 120 kVp; 150 to 200 mA; and section thickness, 1.25 mm for transverse images. CT scanning was obtained 90 seconds once again after the administration of contrast material (100 mL of iopamidol [Iomeron 300 (Bracco, Milan, Italy)]) at a rate of 1.5 mL/s using a power injector. This was followed by a 20-cm3 saline flush at a rate of 1.5 mL/s. Noncontrast data were reconstructed using a bone algorithm whereas contrast imaging data were reconstructed with soft tissue algorithms. Image data were interfaced to a picture archiving and communication system (Path-Speed or Centricity 2.0; GE Healthcare, Mt. Prospect, IL) displaying all data on two monitors (1536 × 2048 matrix, 8-bit viewable grayscale, 60-foot-lambert luminescence). The monitors were adapted to view both mediastinal (width, 400 HU; level, 20 HU) and lung (width, 1500 HU; level, −700 HU) window images. Two radiologists (3 years of experience in radiology and 15 years in thoracic radiology, respectively) reviewed CT scans to determine tumor location, necrosis, cavitation, obstructive pneumonitis/atelectasis, pleural effusion, emphysema, interstitial lung abnormalities (ILAs), and “heterogeneity” and “enhancement ratio” of the tumor.

**Fig 2:**
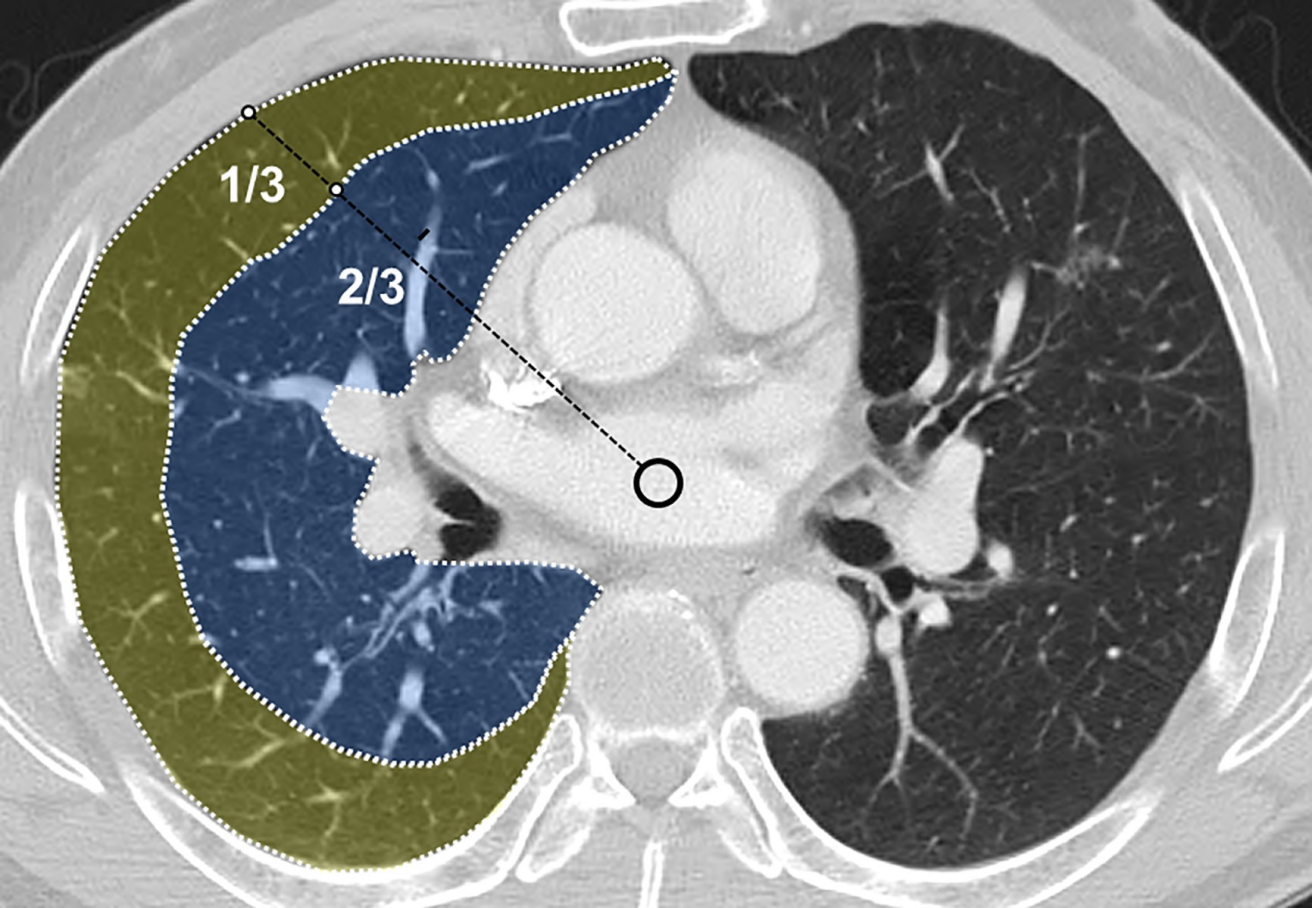
Diagram showing how tumor location was determined. The center (circle) of the tracheal lumen or imaginary downward extension of the trachea was determined at the level of the tumor’s maximum diameter on the axial scan. Then an imaginary border separating the peripheral one-third and central two-thirds was drawn. This border represented the boundary dividing peripheral and central tumors.

**Fig 3:**
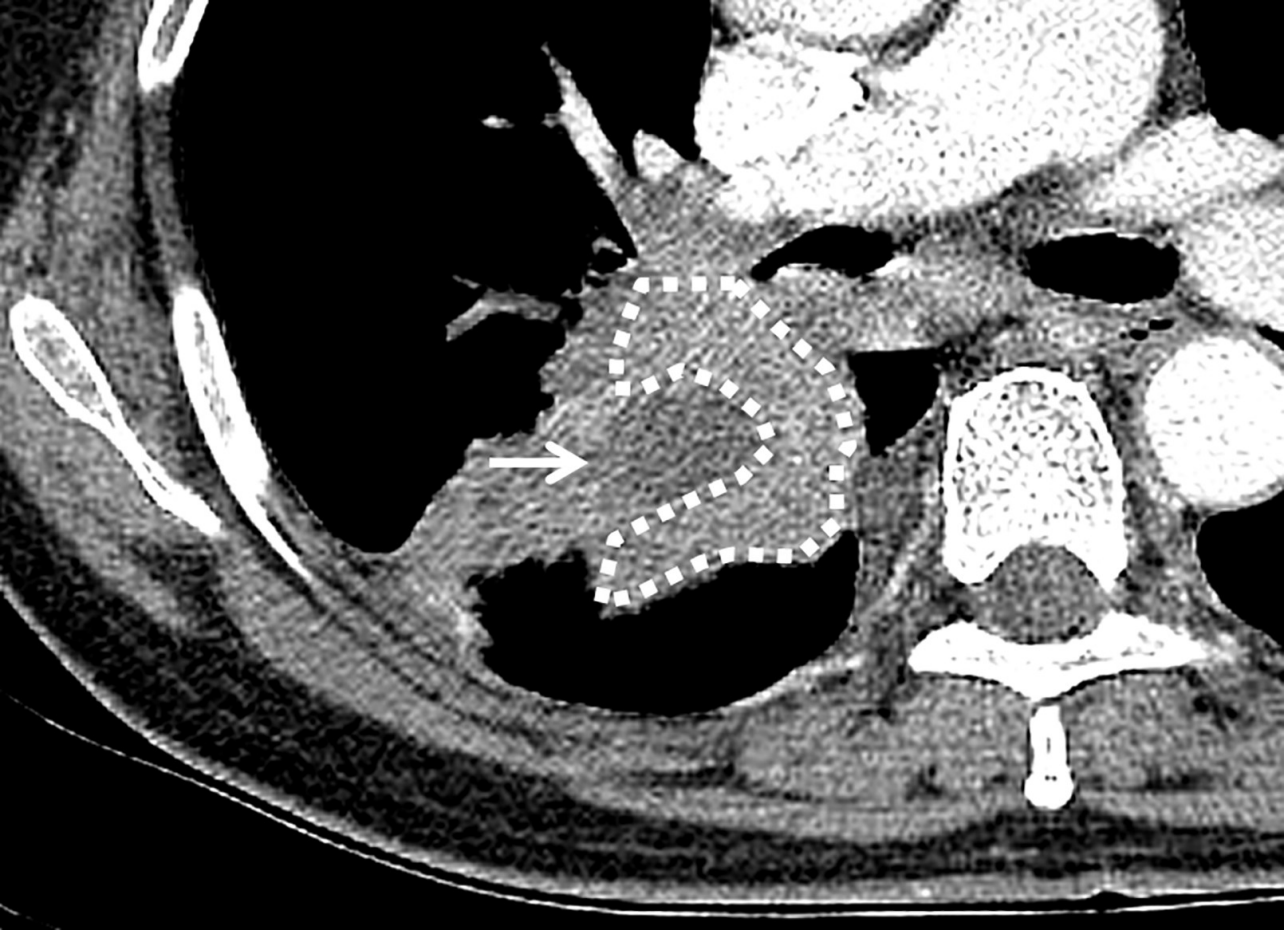
Placement of ROIs within the tumor. Regions of interest were drawn on the outline of the enhancing portion of the tumor (Polygon with a dashed margin). Areas showing necrosis (arrow) or cavitation were excluded from the ROI. ‘Heterogeneity’ was the standard deviation of CT values of the tumor, and ‘enhancement ratio’ was the mean CT value divided by that of the pectoralis muscle.

Firstly, the tumor margin was defined on chest CT image, referring to PET/CT when discriminating it from obstructive pneumonitis/atelectasis. ‘Central’ location was defined as presence of the tumor center at the inner two thirds of the lung field (Fig 2). Intratumoral area larger than 5 mm in diameter with a CT value between 10 and 30 HU and no increment more than 10 HU after contrast administration was considered necrotic. All measurable CT values were derived from the drawn region of interest (ROI) (Fig 3), for which a circular-shaped ROI was located within the tumor as large as possible, also avoiding necrotic areas. ‘Heterogeneity’ indicated the standard deviation of CT values in the ROI. The ratio of mean CT values of a tumor to that of the pectoralis muscle represented the ‘enhancement ratio’. CT-determined ILAs were defined as nondependent changes affecting more than 5% of any lung zone, including reticular or ground-glass abnormalities, diffuse centrilobular nodularity, nonemphysematous cysts, honeycombing, or traction bronchiectasis [10]. The presence of emphysema and ILAs were evaluated qualitatively. We decided to count only the more predominant one for feasible statistical analysis if both were present. After independent review, inter-reader discrepancies were identified and the final decision was made according to consensus.

#### Clinical and histopathologic data analysis

We obtained all data from the electronic medical record system at our institution. Age at diagnosis, sex, and preoperative laboratory results including pulmonary function, serum carcinoembryonic antigen (CEA) levels, and bronchoscopy reports were included. Pulmonary function test results included FEV1 (%) and FEV1/FVC (%). Direct tumor visualization, mucosal nodularity, cancer bleeding, or extrinsic compression on bronchoscopy was considered positive. Last follow-up date, survival status, and time interval from surgery to development of local recurrence, distant metastasis, or death was recorded. Whether the patient underwent adjuvant therapy was documented. If there was concomitant cancer elsewhere, disease-free survival was determined for lung cancer only.

The mode of surgery was categorized as pneumonectomy or subpneumonectomy. TNM descriptors of all patients were re-evaluated and determined according to the AJCC 7^th^ edition [11]. The differentiation state of the tumor was recorded. Lymph node ratio (LNR), representing the number of nodes with positive tumor cells divided by the number of all resected nodes, was measured.

### Statistical analysis

Reliability and reproducibility of CT features was estimated by comparing the intraclass correlation coefficient (ICC) values between the two radiologists. Univariate and multivariate Cox regression analysis were performed to identify the clinicopathologic and radiologic variables correlated with overall survival (OS) and disease-free survival (DFS). Overall survival was defined as the interval between the date of diagnosis and date of death by any cause. Both were estimated using the Kaplan–Meier method. The variables for multivariate analysis were selected according to the backward selection method after univariate analysis. Differences between central and peripheral cancers were compared in terms of clinical, radiologic, and histopathologic variables. Survival status with OS and DFS was also compared between the two categories of cancers. Comparison of continuous variables was performed using the Mann-Whitney test, and categorical variables were compared using Fisher’s exact test. A kernel-Epanechnikov smoothing method at three-month intervals was used to estimate monthly hazard rates [12]. Competing risk analysis was used to assess the cumulative incidence rate of recurrence in each subgroup, and Gray’s test was used to compare the subgroups [12]. Death events were regarded as competing risks when analyzing recurrence. P-values less than 0.05 were considered statistically significant for all statistical tests. Statistical analyses were carried out using SAS software (Version 9.4, SAS institute, Cary, NC) and R (R 3.3.2, Vienna, Austria; http://www.R-project.org).

## Results

### Reliability and reproducibility of CT analysis

The range of ICC values was 0.774 to 0.999, with mean a value of 0.897, representing a substantially high reliability level of agreement.

### Patient characteristics and clinical outcomes

Detailed demographics of the included 382 patients are listed in Table 1. Three patients had both emphysema and ILAs, with emphysema being dominant; they were not categorized as a separate group as the number was too small for statistical analysis. Tumor location was classified as ‘central’ in 230 patients (60.2%). A bronchoscopic abnormality indicating primary tumor or hilar adenopathy was present in 239 patients (62.6%). LNR was zero in most of the patients (n = 356, 93.2%) and no one had pathologic N3 disease. Forty-two patients with clinically equivocal status for N2 disease had histopathologic N2 disease on surgical exploration. The median follow-up period was 56.2 months. During the follow-up period, 159 patients (41.6%) died and 199 patients (52.1%) experienced local recurrence or distant metastasis.

**Table 1:** Patient characteristics.

**Age at diagnosis (years)** ^**†**^		66 years old [60, 71]
**Sex**		370 male, 12 female
**Radiologic variables**		
Central vs. peripheral		230 (60.2%) vs. 152 (39.8%)
Obstructive pneumonitis/atelectasis	Yes	176 (46.1%)
	No	206 (53.9%)
Enhancement ratio*		1.17 ± 0.36
Heterogeneity (HU)*		28.57 ± 13.73
Necrosis or cavitation	None	246 (64.4%)
	Necrosis	112 (29.3%)
	Cavitation	24 (6.3%)
CT-determined lung disease	Normal	282 (73.8%)
	Emphysema	81 (21.2%)
	Interstitial lung abnormalities	19 (5.0%)
**Clinical variables**		
Bronchoscopic detectabililty	Yes	239 (62.6%)
	No	143 (37.4%)
FEV1 (% PRED)*		89.7 ± 17.7
FEV1/FVC (%)*		53.8 ± 11.4
Serum CEA (ng/ml)*		4.52 ± 21.65
Surgery	Pneumonectomy	55 (14.4%)
	Subpneumonectomy	327 (85.6%)
Adjuvant therapy	Chemotherapy	228 (59.7%)
	Radiation therapy	111 (29.1%)
	None	134 (35.1%)
**Histopathologic variables**		
Pathologic T status	T1	71 (18.6%)
	T2	231 (60.5%)
	T3	72 (18.9%)
	T4	8 (2.1%)
Pathologic N status	N0	245 (64.1%)
	N1	95 (24.9%)
	N2	42 (11.0%)
Differentiation	WD	38 (10.0%)
	MD	254 (66.4%)
	PD/DD	90 (23.6%)
Lymph node ratio*		0.03 ± 0.14

CEA, carcinoembryonic antigen; DD, dedifferentiated; FEV1, forced expiratory volume in a second; FVC, forced vital capacity; MD, moderately differentiated; PD, poorly differentiated; WD, well-differentiated. Except where indicated, numbers in parentheses are percentages.

^†^Data are median values [1^st^ quartile, 3^rd^ quartile].

*Data are mean values ± standard deviation.

### Survival analysis

Estimated median OS was 105.1 months and DFS was 67.6 months. On univariate analysis **(**Table 2), poorer OS was correlated with increasing age (*p*<0.001), CT-determined ILAs (*p*<0.001), increasing heterogeneity (*p*<0.001), and pathologic N2 stage (*p* = 0.004) compared to those without nodal metastasis. A correlation was also observed between tumor cavitation and poor OS (*p* = 0.039).

**Table 2:** Univariate Cox regression analysis between variables and overall survival and disease-free survival.

	Overall survival (OS)		Disease-free survival (DFS)	
Variables	*P*-value	HR [95% CI]	*P*-value	HR [95% CI]
Age	**< 0.001**	**1.041 [1.020–1.063]**	**0.004**	**1.027 [1.009–1.046]**
Sex (male vs. female)	0.786	0.884 [0.363–2.155]	0.895	0.947 [0.420–2.134]
Radiologic variables				
Location (peripheral vs. central)	0.503	1.115 [0.812–1.530]	0.724	1.052 [0.792–1.398]
Obstructive pneumonitis/atelectasis	**0.012**	**0.664 [0.484–0.913]**	**0.041**	**0.746 [0.562–0.988]**
Enhancement ratio	0.706	0.921 [0.599–1.414]	0.845	0.963 [0.657–1.410]
Heterogeneity (HU)	**< 0.001**	**1.016 [1.007–1.025]**	0.166	1.009 [0.996–1.021]
Necrosis	0.106	1.328 [0.942–1.873]	0.058	1.342 [0.990–1.820]
Cavitation	**0.039**	**1.775 [1.029–3.063]**	0.237	1.383 [0.808–2.367]
CT-determined lung disease	**< 0.001**		**< 0.001**	
Emphysema	**0.048**	**1.452 [1.004–2.099]**	0.083	1.338 [0.962–1.861]
Interstitial lung abnormalities	**< 0.001**	**6.208 [3.680–10.47]**	**< 0.001**	**4.374 [2.624–7.290]**
Pleural effusion	0.619	1.254 [0.515–3.056]	0.833	0.909 [0.374–2.209]
Clinical variables				
Bronchoscopic detectability	0.110	0.773 [0.563–1.060]	0.468	0.899 [0.676–1.197]
FEV1 (% PRED)	0.508	1.003 [0.994–1.012]	0.510	0.997 [0.989–1.005]
FEV1/FVC (%)	0.129	1.011 [0.997–1.025]	0.665	1.003 [0.990–1.015]
Serum CEA	0.585	0.998 [0.989–1.006]	0.403	0.996 [0.987–1.005]
Mode of surgery (subpneumonectomy vs. pneumonectomy)	0.235	0.778 [0.514–1.177]	0.207	0.786 [0.541–1.142]
Any adjuvant therapy (+)	0.696	1.069 [0.765–1.494]	0.053	1.354 [0.996–1.842]
Histopathologic variables				
Pathologic T stage	0.086		0.158	
T2 vs. T1	0.364	1.235 [0.783–1.950]	0.498	1.147 [0.772–1.704]
T3 vs. T1	**0.024**	**1.816 [1.082–3.049]**	0.060	1.555 [0.982–2.464]
T4 vs. T1	0.216	1.841 [0.699–4.847]	0.143	1.924 [0.802–4.614]
Pathologic N stage	**0.013**		0.075	
N1 vs. N0	0.192	1.277 [0.884–1.845]	0.305	1.190 [0.854–1.657]
N2 vs. N0	**0.004**	**1.920 [1.233–2.989]**	**0.027**	**1.592 [1.055–2.402]**
Differentiation	0.480		0.420	
MD vs. WD	0.875	1.043 [0.616–1.768]	0.952	0.986 [0.615–1.580]
PD/DD vs. WD	0.388	1.290 [0.723–2.301]	0.437	1.228 [0.732–2.062]
Lymph node ratio	0.525	1.365 [0.523–3.564]	0.844	1.098 [0.434–2.777]

CEA, carcinoembryonic antigen; DD, dedifferentiated; FEV1, forced expiratory volume in a second; FVC, forced vital capacity; MD, moderately differentiated; PD, poorly differentiated; WD, well-differentiated. Data with a *p*-value less than or equal to 0.05 are indicated by bold text.

**Fig 4:**
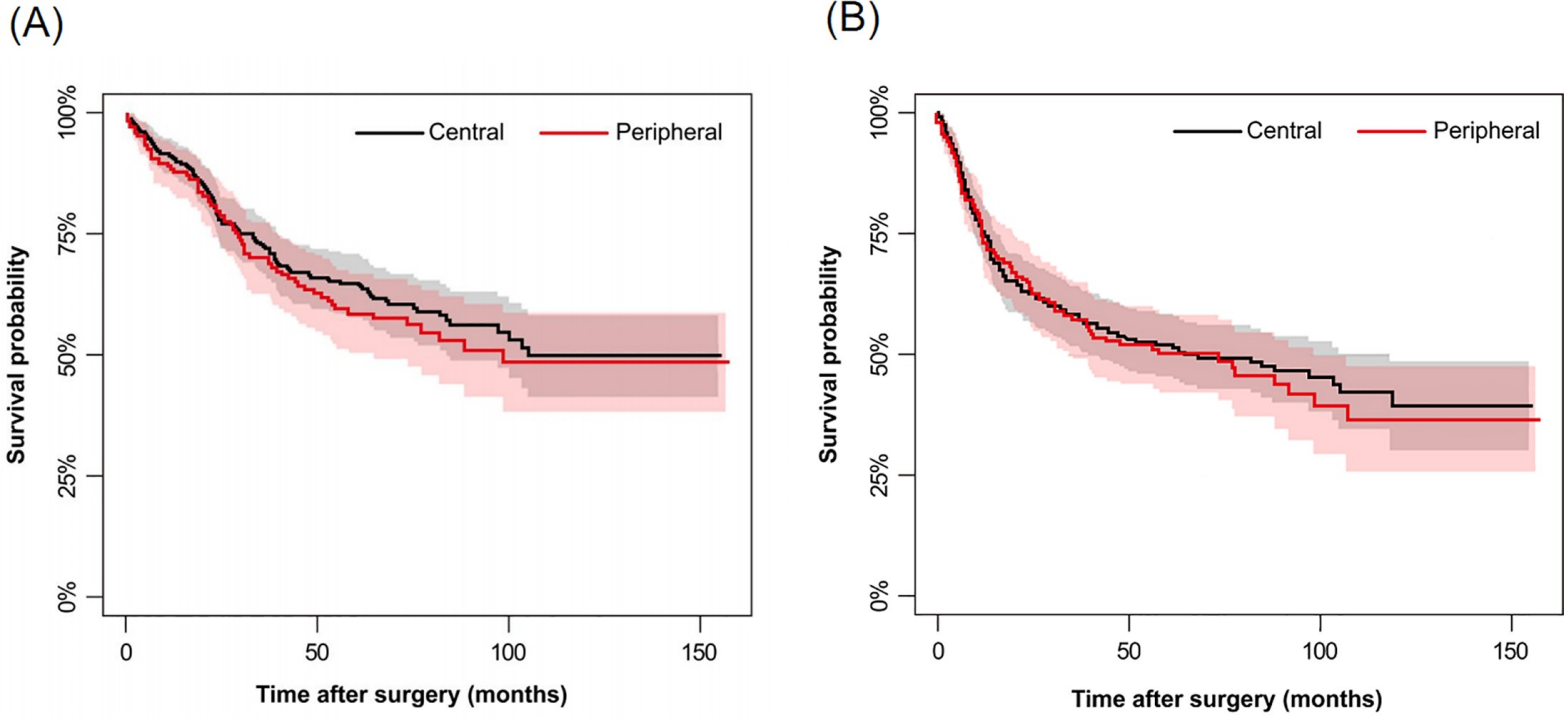
Comparison of calculated OS and DFS using the Kaplan-Meier method according to tumor location. Overall survival (A) and disease-free survival (B) curves are shown according to tumor location. Survival rates were calculated by Kaplan-Meier method. Standard errors were calculated using Greenwood's formula. No significant difference was observed between the two groups. Gray and pink areas represent the 95% confidence intervals of the black and red plots, respectively.

A similar correlation was found between poorer DFS and increasing age (*p* = 0.004), CT-determined ILAs (*p*<0.001), and histopathologic N2 disease (*p* = 0.027). Adjuvant therapy showed a weak correlation to poor DFS (*p* = 0.053). Preoperative FEV1 and FEV1/FVC had no impact on OS (*p* = 0.508 and 0.129) or DFS (*p* = 0.510 and 0.665). Interestingly, obstructive pneumonitis/atelectasis was correlated with both favorable OS (*p* = 0.012) and DFS (*p* = 0.041), while OS and DFS did not vary according to tumor location (*p* = 0.503 and 0.724, respectively) (Fig 4).

On multivariate analysis (Table 3), increasing age (*p* = 0.002), CT-determined ILAs (*p*<0.001), and nodal metastasis (*p* = 0.009 for pN1 vs. N0, *p*<0.001 for pN2 vs. pN0) were correlated with poor OS, while age (*p* = 0.048) and CT-determined ILAs (*p*<0.001) were correlated with poor DFS. Previous adjuvant chemoradiation again showed correlation to poor DFS (*p* = 0.040).

**Table 3:** Multivariate Cox regression analysis between variables and overall survival and disease-free survival.

	Overall survival (OS)		Disease-free survival (DFS)	
Variables	*P*-value	HR [95% CI]	*P*-value	HR [95% CI]
Age	**0.002**	**1.036 [1.013–1.059]**	**0.048**	**1.019 [1.000–1.039]**
Radiologic variables				
CT-determined lung disease				
Emphysema	0.076	1.409 [0.965–2.058]	0.263	1.214 [0.865–1.704]
Interstitial lung abnormalities	**< 0.001**	**6.214 [3.611–10.66]**	**< 0.001**	**4.381 [2.599–7.385]**
Clinical variables				
Any adjuvant therapy (+)			**0.040**	**1.387 [1.015–1.897]**
Histopathologic variables				
Pathologic N stage				
N1 vs. N0	**0.009**	**1.660 [1.134–2.428]**		
N2 vs. N0	**< 0.001**	**2.182 [1.395–3.412]**		

CEA, carcinoembryonic antigen; DD, dedifferentiated; FEV1, forced expiratory volume in a second; FVC, forced vital capacity; MD, moderately differentiated; PD, poorly differentiated; WD, well-differentiated. Data with a *p*-value less than or equal to 0.05 are marked with bold text.

### Central versus peripheral SCC

The differences in radiologic, clinical, and histopathologic aspects between central and peripheral SCCs are detailed in Table 4. The enhancement ratio was marginally higher in central cancers (*p* = 0.051), while no difference in heterogeneity was found (*p* = 0.350). Cavitation and necrosis were more frequently observed in peripheral cancers (*p*<0.001). Patients with central cancers had obstructive pneumonitis/atelectasis more frequently (*p*<0.001). CT-determined lung disease was more prevalent in peripheral cancers (*p*<0.001), especially ILAs (*p*<0.001), and was observed in 17 of 152 peripheral SCC patients and in 2 of 230 central SCC patients.

**Table 4:** Differences between central and peripheral SCCs.

	Central (n = 230)	Peripheral (n = 152)	*P*-value
Mean age (years)*	63.5 ± 7.95	67.1 ± 7.64	< 0.001
**Sex**			0.553
Male	224 (97.4%)	146 (96.1%)	
Female	6 (2.6%)	6 (3.9%)	
**Radiologic variables**			
Obstructive pneumonitis/atelectasis (+)	166 (72.2%)	10 (6.6%)	< 0.001
Enhancement ratio*	1.21 ± 0.35	1.12 ± 0.37	0.051
Heterogeneity (HU)*	28.97 ± 13.91	27.97 ± 13.48	0.350
Necrosis or cavitation			< 0.001
Necrosis	57 (24.8%)	55 (36.2%)	
Cavitation	6 (2.6%)	18 (11.8%)	
CT-determined lung disease			< 0.001
None	184 (80.0%)	98 (64.5%)	
Emphysema	44 (19.1%)	37 (24.3%)	
Interstitial lung abnormalities	2 (0.9%)	17 (11.2%)	
Pleural effusion	6 (2.6)	6 (2.6%)	1.000
**Clinical variables**			
Bronchoscopic detectability (+)	202 (87.8%)	37 (24.3%)	< 0.001
FEV1 (% PRED)*	87.1 ± 17.1	93.5 ± 17.9	0.002
FEV1/FVC (%)*	52.9 ± 11.8	55.2 ± 10.6	0.019
Serum CEA (ng/ml)*	4.07 ± 22.9	5.21 ± 19.7	0.109
Mode of surgery			< 0.001
Pneumonectomy	53 (23.0%)	2 (1.3%)	
Subpneumonectomy	177 (77.0%)	150 (98.7%)	
Adjuvant treatment	160 (69.6%)	88 (57.9%)	0.022
**Histopathologic variables**			
Pathologic T stage			0.004
T1	30 (13.0%)	41 (27.0%)	
T2	147 (63.9%)	84 (55.3%)	
T3	46 (20.0%)	26 (17.1%)	
T4	7 (3.0%)	1 (0.7%)	
Pathologic N stage			< 0.001
N0	125 (54.4%)	120 (79.0%)	
N1	74 (32.2%)	21 (13.8%)	
N2	31 (13.5%)	11 (7.2%)	
Differentiation			0.065
WD	26 (11.3%)	12 (7.9%)	
MD	159 (69.1%)	95 (62.5%)	
PD/DD	45 (19.6%)	45 (29.6%)	
Lymph node ratio*	0.03 ± 0.15	0.02 ± 0.12	0.169

CEA, carcinoembryonic antigen; DD, dedifferentiated; FEV1, forced expiratory volume in a second; FVC, forced vital capacity; MD, moderately differentiated; PD, poorly differentiated; WD, well-differentiated. Except where indicated, numbers in parentheses are percentages. Comparisons between groups were performed using the Mann-Whitney test for continuous variables and Fisher’s exact test for categorical variables.

*Data are mean values ± standard deviation.

With regard to clinical variables, patients with central SCCs were younger at diagnosis than those with peripheral cancers (*p*<0.001). Bronchoscopy found more abnormalities for central cancers (*p*<0.001), which exhibited more nodal metastasis (*p*<0.001). For peripheral cancers, surgery was carried out in a localized fashion rather than via pneumonectomy (*p*<0.001). Adjuvant chemotherapy and radiation therapy was more frequent in patients with central cancers (*p* = 0.022). Serum CEA levels did not differ between the two groups (*p* = 0.109).

**Fig 5:**
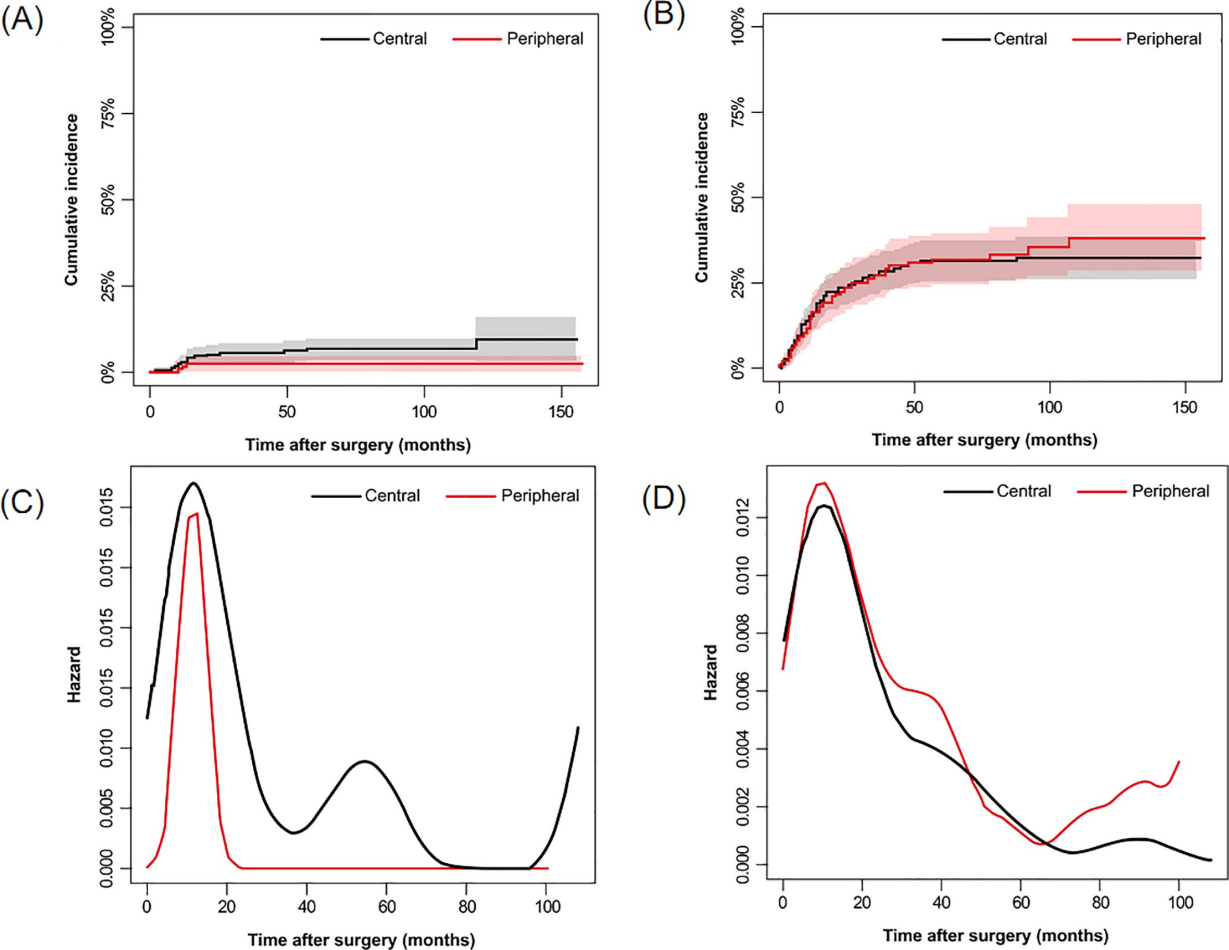
**Comparison of the Cumulative Incidence Rate of Recurrence and Metastasis (A and B) and Recurrence/Metastasis Hazard Rate (C and D) according to Tumor Location.** The rate of local recurrence is shown in A and C, and the rate of distant metastasis is shown in B and D. Central tumors had higher peaks on the local recurrence curve, while peripheral tumors were at higher risk of developing distant metastasis 60 months after surgery. Gray and pink areas represent the 95% confidence intervals of the black and red plots in A and B.

Differences in recurrent dynamics between central and peripheral tumors were noted. Tumor recurrence occurred in about one-third of all patients (n = 139, 36.4%), with distant metastasis in 125 patients, local recurrence in 25, and both in 11. Most patients who only experienced local recurrence had a central tumor (12/14, 85.7%). The hazard rate curve for local recurrence of central cancers had two peaks at 15 months and 60 months after surgery, but peripheral cancers exhibited almost zero risk for the same event 24 months after surgery (Fig 5). However, the rate for distant metastasis was higher 60 months after surgery for peripheral cancers, not central cancers.

## Discussion

Lung SCCs are challenging to manage because SCC patients tend to be older than those with ADCs [13], and this age difference is associated with a higher possibility of comorbidities. However, research regarding the prognostic factors of SCC, which would enable more effective management, remains sparse. Our study focused on determining preoperative radiologic prognosticators in a relatively large number of patients with lung SCC, as well as comparing the clinical and prognostic aspects of central and peripheral SCCs.

A previous study defined the term of ILA as specific patterns of increased density on CT scans [10]. ILAs may be associated with a greater risk of all-cause mortality, suggesting they may represent a subclinical and/or early form of fibrosis, particularly despite being often undiagnosed and asymptomatic [14,15]. They are also a notorious risk factor for developing SCC and lead to poorer outcomes, as in emphysema [16,17]. Our study found that baseline ILAs on CT were trustworthy predictors for poor prognosis. Furthermore, background lung disease had a stronger correlation with poor prognosis than preoperative pulmonary function tests. Patients with combined pulmonary fibrosis and emphysema (CPFE) may not exhibit any preoperative pulmonary function abnormalities [18], but may manifest very poor outcomes after surgery, even in patients with early-stage disease [19,20]. Thus, ILAs and emphysema should be considered important biomarkers in treatment planning.

Histological tumor necrosis is a known biomarker predicting poor prognosis in lung SCCs [21–24], but tumor necrosis on CT scans had no impact in our study. Instead, the heterogenous enhancement reflecting intratumoral neovascularization was correlated with poor OS. Both heterogeneous enhancement and small necrotic foci in the ROI contribute to an increased heterogeneity value due to diverging CT values within the ROI, which may explain the significant relationship between heterogeneity and OS.

There were several differences between central and peripheral cancers in both clinical and radiologic aspects, while no difference was observed in overall outcomes. The median age of patients with central cancers was younger (*p*<0.001, 63.5 vs. 67.1) than those with peripheral cancers, as in a previous study [9]. Definite N stage differed significantly according to tumor location, with more prevalent positive nodal metastasis in central cancers. This finding is in line with studies reporting that central cancers have a lower incidence of N0 disease and lymphovascular invasion than peripheral ones [9,25]. The presence of obstructive pneumonitis/atelectasis, which was more frequent with central cancers compared to peripheral ones (72.2% vs 6.6%), had a protective effect for survival. This finding is paradoxical, given that obstructive pneumonitis/atelectasis generally suggests a higher tumor burden. However, central tumors simultaneously had both more obstructive pneumonitis/atelectasis and a lower prevalence of CT-determined ILAs than peripheral tumors. This difference also explains the similar OS and DFS of both groups, highlighting the importance of CT-determined lung disease. Different recurrence dynamics of central and peripheral SCCs was also revealed by similar methodology in a recent study [12], implying that varied follow-up strategies may be needed for some SCCs according to their location. Further research is needed to confirm these patterns and implement corresponding tailored cancer treatment.

We classified tumor location by measuring the distance between the tumor center and pleural interfaces on axial CT scan images, and not by its real branching order off the main airway [8]. We adopted this method because determining the real number of the branching order is not possible in actual practice. Our method might be incorrect, particularly for cancers located in the apical or basal lung and explain the lack of meaningful correlation between tumor location and prognosis.

LNR can be a prognostic variable in addition to TNM staging in NSCLC [26,27]. As many nodes as possible should be examined to calculate the LRN accurately without missing any metastatic nodes. This approach allows for reduced likelihood of local recurrence and accurate prediction of which patients will benefit from adjuvant radiotherapy. In this context, our study sought to explore the relationship between LNR and OS and DFS, but we were unable to find any significant relationship because LNR was zero in most of the patients. Here, the weak correlation between adjuvant therapy and poor DFS could be partly due to biased selection for adjuvant treatment among patients with advanced stages.

In addition to efforts to identify patients at high risk for developing recurrence, thoughtful surveillance after curative-intent treatment and timely management of recurrence represent additional components for improving treatment outcomes. To do so requires understanding the dynamics of recurrence according to tumor location, and our study demonstrated substantially intriguing results. In terms of central SCCs, the hazard rate of recurrence peaked approximately 55 months once more following the first peak at 18 months after resection, in contrast with peripheral SCCs. This finding justifies an emphasis on intensive surveillance during this period, particularly in cases of central SCCs.

There are several limitations to this study. First, this was a retrospective study performed at a single center. As a result, where technical factors related to CT scan such as reconstruction kernel, slice thickness or interscanner differences may cause considerable measurement variability, we carefully set inclusion criteria for sufficient image quality.

Second, this study dealt with a group of patients who underwent treatment between 2003 and 2012. Treatment regimens may have changed during that period, which could have affected clinical outcomes. Smoking history, a significant risk factor in lung cancer, was not evaluated. In addition, another experimental approach such as immunochemical or genomic data and consequent integrated analysis is needed to better understand tumorigenesis or heterogeneity in SCCs.

In conclusion, our study suggests that CT-determined ILA is an important biomarker predicting outcomes in lung SCCs. Several differences were also found between central and peripheral SCCs, suggesting differences in the physiology of carcinogenesis, as both groups manifested different recurrence dynamics.

## Supporting information

S1 File All patients information used for analysis. (XLSX)

## Abbreviations

ADC: adenocarcinoma
AJCC: American Joint Committee on Cancer
CEA: carcinoembryonic antigen
CT: computed tomography
FDG: fluorodeoxyglucose
FEV1: forced expiratory volume in a second
FVC: forced vital capacity
ILA: interstitial lung abnormality
LNR: lymph node ratio
NSCLC: non-small cell lung cancer
PET: positron emission tomography
PFT: pulmonary function test
SCC: squamous cell carcinoma
SUVmax: maximum standardized uptake value
